# Cross-Domain Association in Metacognitive Efficiency Depends on First-Order Task Types

**DOI:** 10.3389/fpsyg.2018.02464

**Published:** 2018-12-04

**Authors:** Alan L. F. Lee, Eugene Ruby, Nathan Giles, Hakwan Lau

**Affiliations:** ^1^Department of Applied Psychology, Lingnan University, Hong Kong, Hong Kong; ^2^Department of Psychology, University of California, Los Angeles, Los Angeles, CA, United States; ^3^Brain Research Institute, University of California, Los Angeles, Los Angeles, CA, United States; ^4^Department of Psychology, University of Hong Kong, Hong Kong, Hong Kong; ^5^The State Key Laboratory of Brain and Cognitive Sciences, University of Hong Kong, Hong Kong, Hong Kong

**Keywords:** metacognition, 2AFC task, domain-general, domain-specific, behavioral task

## Abstract

An important yet unresolved question is whether or not metacognition consists of domain-general or domain-specific mechanisms. While most studies on this topic suggest a dissociation between metacognitive abilities at the neural level, there are inconsistent reports at the behavioral level. Specifically, while McCurdy et al. (2013) found a positive correlation between metacognitive efficiency for visual perception and memory, such correlation was not observed in Baird et al. (2013). One possible explanation for this discrepancy is that the former included two-alternative-forced choice (2AFC) judgments in both their visual and memory tasks, whereas the latter used 2AFC for one task and yes/no (YN) judgments for the other. To test the effect of task on cross-domain association in metacognitive efficiency, we conducted two online experiments to mirror McCurdy et al. (2013) and Baird et al. (2013) with considerable statistical power (*n* = 100), and replicated the main findings of both studies. The results suggest that the use of task could affect cross-domain association in metacognitive efficiency. In the third experiment with the same sample size, we used YN judgments for both tasks and did not find a significant cross-domain correlation in metacognitive efficiency. This suggests that the cross-domain correlation found in McCurdy et al. (2013) was not simply due to the same task being used for both domains, and the absence of cross-domain correlation in Baird et al. (2013) might be due to the use of YN judgments. Our results highlight the importance of avoiding confusion between 2AFC and YN judgments in behavioral tasks for metacognitive research, which is a common problem in many behavioral studies.

## Introduction

Metacognition is an important cognitive ability that enables us to monitor and regulate our own mental processes and task performance. In experiments, one way to quantify metacognitive sensitivity is to assess the trial-by-trial correspondence between confidence judgments and accuracy in behavioral tasks. An important question that remains unclear is whether the metacognition underlying different processing domains, such as metacognition for visual perception and memory, depend on distinct, domain-specific neurocognitive mechanisms, or on a single, domain-general system that supports metacognition for all mental faculties.

Findings from recent neurophysiological studies (e.g., Baird et al., 2013, 2015; McCurdy et al., 2013; Fleming et al., 2014; Morales et al., 2018) are largely consistent: they found that distinct brain regions were involved when one performed metacognitive tasks in different processing domains (e.g., metacognition for a visual task vs. metacognition for a memory task). This suggests that there exist domain-specific neural mechanisms that support metacognition for a processing domain. Interestingly, findings are, however, somewhat conflicted at the behavioral level.

A commonly-used technique in behavioral studies to address the above domain-general-vs.-domain-specific question is the individual-differences approach. Researchers separately measure metacognitive efficiency in two different processing domains (e.g., vision and memory), and then compute the correlation between the two across individuals. If metacognition is domain-general, the same mechanism should underlie metacognitive responses across domains, and there should be a significant, positive cross-domain association in metacognitive efficiency. Specifically, while McCurdy et al. (2013) reported a positive correlation between metacognitive efficiency for memory (i.e., metamemory) and visual perception, Baird et al. (2013) found no such correlation.

There are a few issues that could explain such discrepancy. The first concerns statistical power. As the two studies had different sample sizes (*n* = 34 for McCurdy et al., 2013 and *n* = 52 for Baird et al., 2013), statistical power may differ between the two studies. Furthermore, the power might be too low in Baird et al.'s case to detect the possibly weak, if any, cross-domain association in metacognitive sensitivity. Therefore, in the present study, we attempted to replicated both experiments with a much larger sample size (*n* around 100) for greater and more similar, comparable level of statistical power.

Another issue is that neither McCurdy et al. (2013) nor Baird et al. (2013) used the same stimulus type between the two tasks. Specifically, both studies used circles with gratings for the visual task, but words for the memory task. In general, this would make it more difficult to compare the metacognitive processes underlying the two tasks. To address this issue, we used the same stimulus type across tasks in the present study.

While the above issues related to statistics and experimental design may have contributed to the inconsistent results, we hypothesize that the most important factor related to examining metacognitive processing was a subtle, yet crucial difference between the two studies, as suggested by Baird et al. (2013) themselves. While McCurdy et al. (2013) required two-alternative forced choice (2AFC) discrimination judgments for both visual and memory tasks, Baird et al. (2013) required 2AFC judgments for the visual task but yes/no (YN) judgments for the memory task. The distinction between a 2AFC task and a YN task is so subtle that many researchers tend to use the two names interchangeably. We clarify the key difference between the two tasks below.

In each trial of a 2AFC task, the participant is presented with two stimuli, either at two different spatial locations simultaneously (the traditional definition for 2AFC) or one after another in succession (some called this a two-interval, forced-choice task or 2IFC). The task is to identify the spatial or temporal arrangements of the two stimuli, e.g., whether the “old word” (that has been previously presented) is on the left and the “new” word is on the right, or vice versa (as was done in McCurdy et al., 2013). The defining feature of a 2AFC task is that *both* stimuli have to be presented.

In each trial of a YN task, the participant answers a binary, “yes-or-no” question about a *single* stimulus in each trial, e.g., whether the presented word is new or old (as was done in Baird et al., 2013). Sometimes, this type of YN task is also known as a two-choice, discrimination task, as the participant discriminates the only presented stimulus between two choices.

Because 2AFC and YN tasks presumably involve rather different perceptual, cognitive, and even decision-making processes, they could affect metacognitive judgments (for more in-depth comparison between the two task types, see section Discussion). This may hinder researchers from obtaining comparable measurements on cross-domain association in metacognitive sensitivity, which could be the main reason for the inconsistent results between the two above-described studies, as well as across many other behavioral findings (e.g., Valk et al., 2016; Sadeghi et al., 2017; Morales et al., 2018).

Therefore, the main goal of the present study was to empirically address the above issues through a series of experiments. We systematically varied the use of tasks (2AFC vs. YN) for different processing domains, namely, vision and memory, and measured the resulting cross-domain association in metacognitive sensitivity. In terms of the use of tasks, Experiment 1 replicated McCurdy et al. (2013) in that both the visual and memory tasks involved 2AFC judgments. Experiment 2 replicated Baird et al. (2013) in that the visual task involved 2AFC judgments while the memory task involved YN judgments. To further examine the possibility that cross-domain association might simply be due to using the same type of task across domains, we used YN judgments for both visual and memory tasks in Experiment 3, which was similar to Fitzgerald et al.'s (2017) study.

At the same time, we addressed the above-mentioned issues about statistical power and stimulus design by conducting an online study (*n* = 100 for each experiment) and using the same stimulus (cluster of circles) for both the visual and memory tasks, which would be different from the designs in McCurdy et al. (2013) and Baird et al. (2013), but could reconcile the inconsistent findings between the two studies.

## Materials and Methods (for all Experiments)

### Subjects

In each of the following experiments, 100 healthy subjects were recruited using Amazon Mechanical Turk's task hosting service. All experiments were conducted through the Internet (using the same service). Eligibility was determined by the subjects and listed in an online advertisement and consent form for the study; in order to take part in the experiment, all subjects were required to have normal or corrected-to-normal vision (e.g., glasses or contact lenses) and no history of any psychiatric or neurological illnesses or seizures. All subjects provided consent to participate. Each subject was compensated $4 for completion of each experiment, with the possibility of receiving a $1 bonus if their performance was higher than that of the previous participant.

### Stimulus

The stimulus for both the visual and memory tasks was a cluster of black-outlined circles with varying sizes against a white background. Positions of circles were randomly determined, with the following constraints: Circles were not allowed to overlap with one another, and the distance between any two circles within the cluster was limited within a certain range, so that circles would not be too far or too close to one another.

### Visual Task

The visual task was an average-size comparison task. Radius of each circle was randomly sampled from a predetermined average number of pixels. This average radius varied according to a staircase procedure described below. In each experiment, each subject completed 120 trials of the visual task.

**Figure 1:**
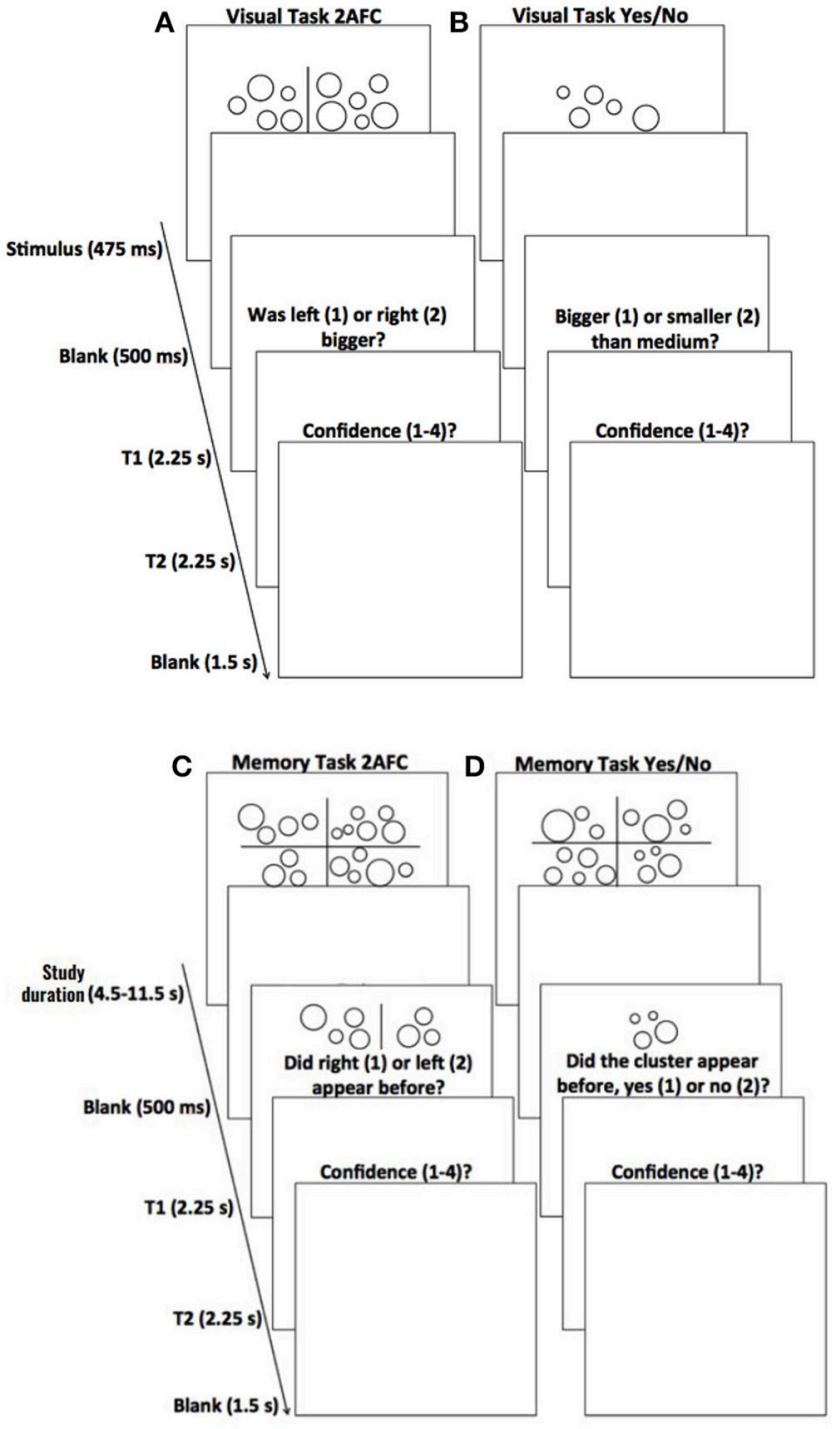
Timeline for individual trials on the various tasks. **(A)** Visual task with 2AFC judgments. Subjects were shown two clusters of circles, followed by a blank screen, and were then prompted to discriminate whether the cluster with (on average) larger circles was on the left or right (T1). They then rated how confident they were on a scale of 1–4 (with 1 being not at all confident and 4 being completely confident) in their discrimination judgment (T2). A blank screen ended the trial. **(B)** Visual task with YN judgments. Each visual task trial requiring YN judgments was identical to trials for the 2AFC visual task, except that subjects were shown only one cluster, and were later prompted to discriminate whether the cluster had (on average) larger circles than a cluster of “medium” size (T1). **(C)** Memory task with 2AFC judgments. Subjects were shown four patterns of circles, followed by a blank screen. Next, two patterns were presented, one from the previous stimulus presentation and one new pattern; participants were instructed to pick the pattern that appeared before (T1). As with the visual tasks, they then gave confidence judgments on a scale of 1–4 (T2). A blank screen ended the trial. **(D)** Memory task with YN judgments. Each memory task trial requiring YN judgments was identical to trials for the 2AFC memory task, except that only one pattern was presented during the discrimination portion of the task and subjects were instructed to determine whether it had appeared on the previous stimulus presentation or not (T1).

In the visual 2AFC task (Figure 1A; for Experiments 1 and 2), subjects were presented with, side by side, two independently generated clusters of circles for 475 ms, followed by a blank screen for 500 ms. Next, subjects were given 2.25 s to indicate which cluster of circles had a larger average size. After this response, subjects were given another 2.25 s to indicate how confident they were in the previous judgment by pressing any number key between 1 and 4, with 1 representing not confident at all and 4 representing extremely confident. After the confidence response, a blank screen was presented for 1.5 s, before the next trial began.

In the visual YN task (Figure 1B; for Experiment 3), subjects were first shown ten different example clusters of circles and were told that they were of “medium” size. Then, in each trial of the YN task, subjects were presented with one cluster of circles and asked to indicate whether circles in this cluster were on average bigger or smaller than those in the medium clusters they had viewed at the beginning. Other stimulus and procedure details were the same as the visual 2AFC task described above.

### Memory Task

The memory task concerned the specific pattern of circles than the average size. Subjects were first presented with four to-be-remembered target patterns, and then completed a memory task on a specific pattern with confident rating afterwards. To control task difficulty in each trial, the “study” duration of the target patterns was varied according to a staircase procedure described below. In each experiment, each subject completed 120 trials of the memory task.

In each trial of the memory 2AFC task (Figure 1C; for Experiment 1), subjects were first presented with four target patterns of circles for a duration ranging from 4.5 s to 11.5 s (i.e., the study duration; controlled by a staircase procedure described below). The four patterns were located at the four quadrants of the screen (see first panel in Figure 1C). A blank screen was then presented for 500 ms. Next, subjects were presented, side by side, with two patterns of circles. One of the two patterns was chosen from the four target patterns that had just been presented, and the other one was new, independently-generated pattern. Subjects were given 2.25 s to indicate which of the two patterns had been previously presented among the four target patterns. Next, subjects were given 2.25 s to indicate the confidence on a scale of 1–4, which was identical to that used in the visual task. A blank screen was then presented for 1.5 s before the next trial began. The immediately-following three trials had the same procedure, except that the four target patterns were not presented again. These four trials constituted a mini-block for the memory 2AFC task.

The memory YN task (Figure 1D; for Experiments 2 and 3) was identical to the memory 2AFC task described above, except for the following. Instead of two patterns (i.e., one from the target, one newly-generated foil), only one pattern was presented. The task was to indicate whether this pattern had been presented among the four target patterns at the beginning of the mini-block.

As in McCurdy et al. (2013) and Baird et al. (2013), all subjects completed both a visual task and a memory task in each of the experiments.

### Staircase Procedure

For both the visual and memory tasks, task difficulty was controlled using *n*-up-*m*-down staircase procedures. In each staircase, difficulty level of the following trial would be adjusted up by one step size (i.e., one unit more difficult) if the subject had consecutively given *n* correct response(s), and adjusted down by one step size (i.e., one unit easier) if the subject had consecutively given *m* incorrect response(s). If the subject had produce less than *n* consecutive correct responses or less than *m* consecutive incorrect responses, difficulty level of the following trial would remained unchanged.

For the visual 2AFC task, difference in the average radius between the two presented clusters was varied to control task difficulty. After each response, the algorithm randomly select between a 2-up-1-down and a 3-up-1-down staircase, and decide the difference in the average radius accordingly. This allowed the 2AFC task performance to converge to an expected value of around 75%. The initial difference in average radius was 6 pixels, and the step size for both increasing and decreasing average radius was 1 pixel. For all trials, the maximum and minimum differences in average radius were fixed at 11 pixels and 1 pixel, respectively.

For the visual YN task, difficulty was controlled by varying the difference in average radius between the presented cluster and the “medium” clusters. The initial difference was 4 pixels, and the step size for both increasing and decreasing average radius was 1 pixel. For all trials, the maximum and minimum differences in average radius were fixed at 7 pixels and 1 pixel, respectively.

For both the memory 2AFC and memory YN tasks, the same staircase procedure was used. Task difficulty was controlled by varying the study duration of the target patterns. At the beginning of each mini-block, this study duration was determined by a variant of a 4-down-2-up staircase procedure with a step size of 500 ms: if the subject had got all four responses correct in the previous mini-block, study duration for the current mini-block would be reduced by 500 ms, making the memory task for the current mini-block more difficult; if the subject had got two or less responses correct in the previous mini-block, study duration for the current mini-block would be increased by 500 ms, making the memory task for the current mini-block easier; otherwise, if the subject had given exactly three out of four correct responses in the previous mini-block, study duration would remain unchanged for the current mini-block. This staircase procedure would allow memory task performance to converge to an expected value of 75%. Study duration for the first mini-block was 8 s for all subjects. The minimum and maximum study duration for all mini-blocks were 4.5 and 11.5s, respectively.

### Additional Measures for Ensuring Active Participation

For both the visual or memory tasks, 5% of trials (i.e., 6 trials for each task in each experiment) were catch trials. The catch trials were inserted to ensure that participants were actively attending to the task, and that they correctly understood the instructions. Each catch trial consisted of a stimulus that would make the task extremely easy. If subjects performed poorly on any two catch trials, they would be told that they had not performed well on some of the easier trials and were asked whether or not they would like to continue the experiment.

Moreover, subjects who did not give responses within the given time (2.25 s) for four consecutive trials were given a warning, indicating that they should pay closer attention to the task, and that they would not be able to continue the experiment if they failed to do so. Following this, if the subject again did not respond in time on four consecutive trials, their participation in the experiment was terminated immediately.

In each experiment, every subject performed both the visual and the memory tasks, with the order of the tasks counterbalanced across subjects. Each experiment took approximately 1 h to complete. All subjects completed all the tasks in one session. As remaining stationary and on task for 1 h might induce boredom or fatigue, subjects were given two short breaks throughout each of the two tasks in order to ensure they retained a sufficient level of comfort and focus.

All stimuli and behavioral tasks were created using JavaScript by utilizing jsPsych. The stimuli in the tasks were scaled off of the size of each subject's computer screen, which ensured that the sizes and positions of all stimuli would be the same for all subjects, despite the fact that computer screen size probably varied from participant to participant.

### Data Analysis

To assess metacognitive sensitivity for each modality, we utilized the bias-free psychophysical measure, meta-d' (Maniscalco and Lau, 2012), which measures how well participants can differentiate between correct and incorrect answers given to the first-order (i.e., visual or memory) task on a trial-by-trial basis (Fleming and Lau, 2014).

In the present study, d' refers to the distance, in standard deviations units, between two stimulus distributions along an internal dimension of perceptual representation (e.g., size) or memory representation (e.g., familiarity). It is a bias-free measure of sensitivity in, for example, discriminating between “larger” and “smaller” clusters of circles in our visual tasks, or between “old” and “new” patterns of circles in our memory tasks. The meta-d' of a participant is the d' that an observer with “optimal metacognition” and the same first-order response bias would require in order to reproduce the participant's metacognitive (or type-2) responses that have been observed in the experiment (Maniscalco and Lau, 2012).

The values of meta-d' and d', together with the values of other relevant parameters (e.g., criteria for type-1 and type-2 decisions) were estimated using the maximum-likelihood estimation (MLE). Formally, let θ be the vector containing the set of parameters to be estimated (including meta-d' and d'). We estimated meta-d' and d' for each participant by finding the θ that maximizes the following likelihood function (Maniscalco and Lau, 2014):

Ltype 2(θ|data)∝∏y,s,rProbθ(conf=y | stim=s,resp=r)ndata(conf=y | stim=s, resp=r)

where conf = y | stim = s, resp = r refers to an experiment trial in which the confidence rating was y for a stimulus of s and response of r, n_data_(conf = y | stim = s, resp = r) refers to the number of such trials in the experiment, and Prob_θ_(conf = y | stim = s, resp = r)refers to the predicted conditional probability of confidence response being y given the stimulus being s and response being r using the parameter values specified in θ based on the standard SDT model. The MLE was carried out using the MATLAB code provided by Maniscalco and Lau (2012; 2014; URL: http://www.columbia.edu/~bsm2105/type2sdt/.

We then divided subjects' meta-d' by their d' scores in the first-order task to obtain meta-d'/d' (M Ratio), a measure of metacognitive efficiency (i.e., a participant's metacognitive sensitivity given a specific level of basic task performance). M Ratio, which controls for the effects of first-order task performance, was also the primary measure of interest in both McCurdy et al. (2013) and Baird et al. (2013); i.e., correlational analyses in these studies assessed the relationship between M Ratio for visual metacognition and metamemory. In the present study, we similarly performed within-subjects correlation analyses to assess relationships between visual metacognition and metamemory.

### Subject Exclusion Criteria

Because the study was conducted online, many factors were beyond our control, leading to extreme or unrealistic values of measurements, particularly in task performance. To prevent these extreme or unrealistic values affect our analyses, subjects were excluded if any of the following criteria were matched.

First, Cook's D (Cook, 1977) was computed for all data points to identify statistical outliers. If the Cook's D for any measure of a subject exceeded the standard threshold recommendation of 4/(n-k-1), where n is the sample size and k is the number of independent variables, that subject's data point was labeled as an outlier, and removed from the analysis. Notably, however, in all experiments we found similar patterns of statistical significance and results, both regardless whether outliers were removed or not (unless otherwise noted in specific cases below).

Second, subjects were excluded if their first-order task performance (visual or memory) or metacognitive sensitivity was not within a reasonable range. Specifically, if d' was too low, the estimate of M Ratio could become unreasonably large and/or unstable (e.g., a meta-d' of 0.1 and a d' of 0.001 would produce an M Ratio of 100). Therefore, the subject was excluded if d' was below 0.5 for either the visual or memory task. Also, because we used a staircase procedure to maintain a relatively stable accuracy of around 75% for both tasks, we assumed subjects produced confidence ratings based on this level of accruacy. If a subject's accuracy deviated too much from this controlled value, it would suggest that his/her confidence ratings may have been made based on a much better or a much worse performance level. This would create an unnecessary source of variability in the data. Therefore, if the percentage correct was 15% below the expected accuracy (i.e., accuracy < 60%) or 15% above the expected accuracy (i.e., accuracy >90%), the subject was excluded. For confidence judgments, a subject would be excluded if meta d' was below 0 for the visual or memory task.

Based on the above exclusion criteria, four, seven, and six subjects were excluded from the analyses in Experiment 1 (*n* = 96), Experiment 2 (*n* = 93), and Experiment 3 (*n* = 94), respectively. In the following Results section, the figures only show data with the above-mentioned outliers removed. For the full set of data showing all 100 participants for each experiment (with outliers labeled) and the corresponding scatterplot of cross-domain M Ratios, please refer to the publicly-available fileset on figshare (doi: 10.6084/m9.figshare.7353266; URL: https://figshare.com/articles/Cross-domain_association_in_metacognitive_sensitivity_depends_on_first-order_task_types/7353266.

## Results

### Experiment 1: 2AFC for Both Visual and Memory Tasks

**Figure 2:**
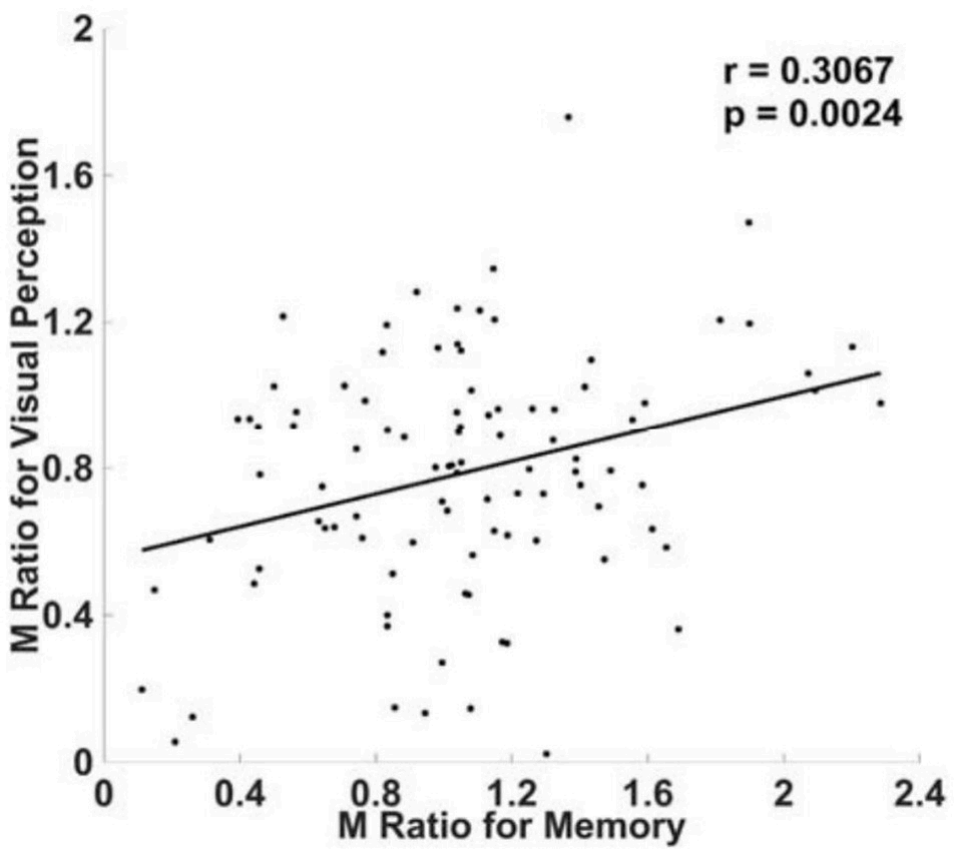
Correlation between visual and memory metacognitive efficiency when when both tasks involved 2AFC. As in McCurdy et al. (2013), we found a significant positive correlation across subjects, between visual and memory metacognitive efficiency when both tasks involved 2AFC rather than YN judgments. Metacognitive efficiency was quantified using M Ratio, a detection theoretic measure of metacognitive efficiency that accounts for fluctuations in task performance (see section Materials and Methods).

As in McCurdy et al. (2013), both tasks in our Experiment 1 included 2AFC judgments (see section Materials and Methods). Ninety-six subjects were included in analyses after subjects exclusion, giving our experiment more statistical power than McCurdy et al. (2013), who included only 34 subjects. We found a significant positive correlation between M Ratio for visual and memory metacognition (*r* = 0.3067, *p* = 0.0024; Figure 2). Of note, this significant correlation was present also without removing influential outliers, with a numerically weaker strength (*r* = 0.2470, *p* = 0.0132).

### Experiment 2: Visual 2AFC Task vs. Memory YN Task

Given our replication of the main finding of McCurdy et al. (2013) in Experiment 1, in our Experiment 2 we changed the discrimination judgments for the memory task from 2AFC to YN judgments while leaving everything else from Experiment 1 unchanged (see section Materials and Methods), and attempted to mimic the set up by Baird et al. (2013). The idea was to see if using this asymmetric design (with one task being 2AFC and the other being YN), one could still observe a significant correlation between metacognitive efficiencies when we have enough subjects.

**Figure 3:**
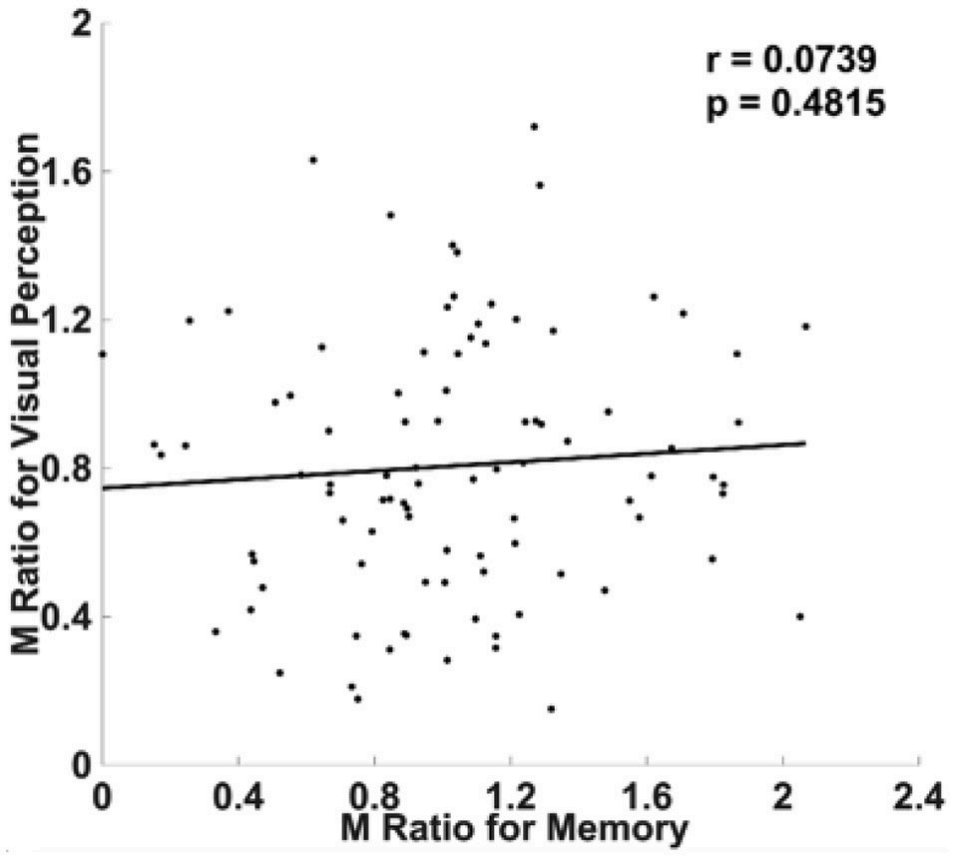
No correlation in metacognitive efficiency when the visual task involved 2AFC judgments and the memory task involved YN judgments. As in Baird et al. (2013), we didn't find a correlation between visual and memory metacognitive efficiency when the visual task involved 2AFC judgments and the memory task involved YN judgments. As in Experiment 1, metacognitive efficiency was quantified using M Ratio (see section Materials and Methods).

Ninety-three subjects were included in analyses after subject exclusion, giving this study more statistical power than Baird et al. (2013), who included 52 subjects. As in Baird et al. (2013), we failed to find a significant correlation between M Ratio for memory and visual metacognition (*r* = 0.0739, *p* = 0.4815; Figure 3). The correlation remained insignificant even without removing the outliers (*r* = 0.01, *p* = 0.9215). In short, we replicated the behavioral result from Baird et al. (2013) even under greater statistical power.

### Across Experiments Analyses

To further probe whether the difference in behavioral results between the two studies can be consistently replicated, we converted Pearson correlation coefficient values for each of the two experiments to z values (as z values, unlike r values, are normally distributed and can therefore be compared) using Fisher's R to Z transformation (Fisher, 1915). A *Z* test(Cohen and Cohen, 1983) revealed that the correlation between M Ratio for visual and memory metacognition decreased significantly from our Experiment 1 to Experiment 2 (*z* = 1.77, one-tailed *p* = 0.0394). Thus, not only did we find different results when using distinct types of discrimination judgments across the two experiments, this difference was also significant under a direct comparison.

### Experiment 3: Visual YN vs. Memory YN

The results of our first two experiments raise the question of what exactly it is about the distinction in judgment type that causes differing results. One possible explanation is that observing a correlation depends on using the same judgment type across tasks. For example, both our Experiment 1 and McCurdy et al. (2013) found significant correlations when using the same type of discrimination judgment for both tasks (2AFC judgments). If the correlations depend on using the same type of judgment across tasks, perhaps we should also find that utilizing YN judgments for both tasks would similarly reveal a significant correlation.

Alternatively, our Experiment 2 and Baird et al. (2013) may have failed to find a correlation because YN judgments are somewhat more complex than 2AFC judgments. 2AFC judgments involve two stimulus alternatives being directly compared based on their “familiarity” (or some other feature reflecting a conscious memory trace), whereas YN judgments involve only one stimulus being present and with no other stimulus directly accessible to be compared. As such, performing a YN judgment requires a stable criterion as to what count as “familiar.” This may place a higher demand on working memory given that the participant likely has to compare the present stimulus to other stimuli that have recently been encountered but are no longer present.

With these two possible explanations in mind, we conducted Experiment 3, using YN judgments for not only the memory task (as in Experiment 2), but also for the visual task (see section Materials and Methods). If significant cross-domain correlation was found, it would suggest that McCurdy et al. (2013) finding might be caused by using the same task type for both domains. If not, it would suggest that the absence of cross-domain correlation in Baird et al. (2013) because of the use of YN judgments.

**Figure 4:**
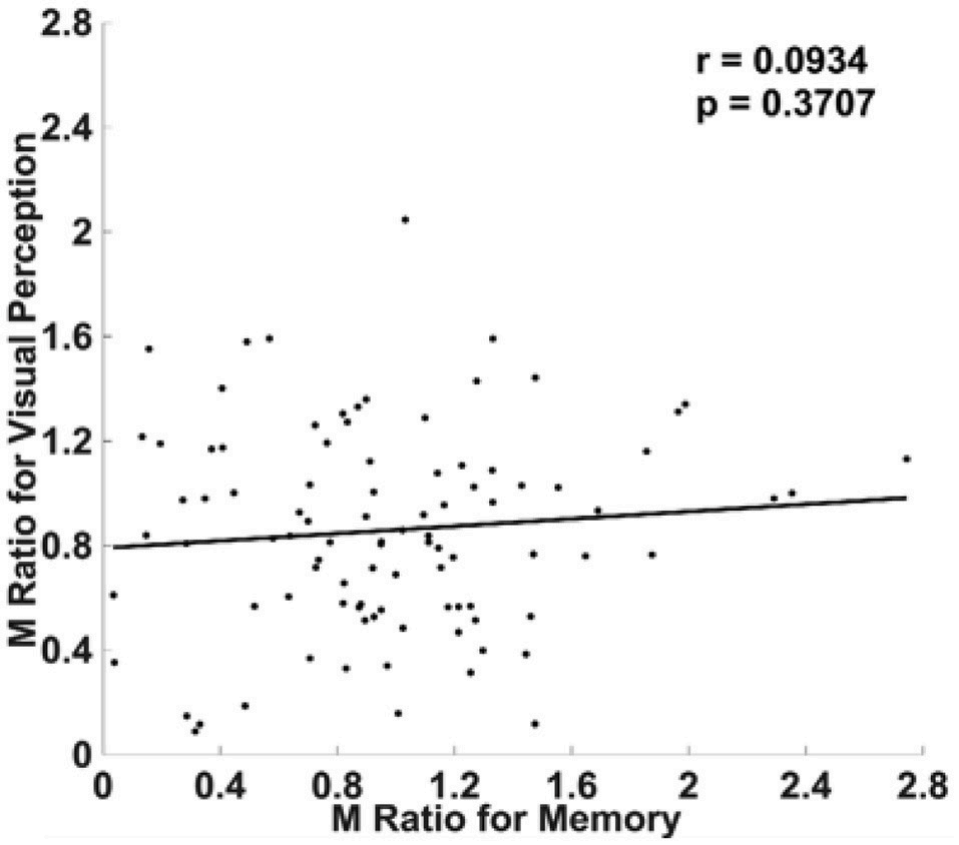
No correlation between visual and memory metacognitive efficiency when both tasks involved YN judgments. We did not find a correlation between visual and memory metacognitive efficiency when both tasks involved YN rather than 2AFC judgments. Metacognitive efficiency was quantified using M Ratio (see section Materials and Methods).

Ninety-four subjects were included in analyses after subject exclusion. We did not find a significant correlation between M Ratio for metamemory and visual metacognition (*r* = 0.0934, *p* = 0.3707; Figure 4). Without removal of outliers, the correlation became slightly stronger, but still remained insignificant (*r* = 0.1025, *p* = 0.3102). Taken alongside the results of our first two experiments, this suggests that the use of a YN task could be the reason for the inconsistent behavioral findings from Rounis et al. (2010) and Bor et al. (2017).

### Further Analyses on “Yes” vs. “No” Responses

If YN judgments are in fact somehow limiting our ability to reveal metacognitive correlations than 2AFC judgments, what exactly is it about the former that makes it so? Above we suggested the possibility that YN judgments might be more demanding in terms of criterion maintenance, and possibly other higher cognitive processes. Further dissecting this proposal, we might ask if this added complexity is true for both “yes” and “no” responses. Of relevance, several previous studies suggest that metacognitive sensitivity is lower for “no” responses than for “yes” responses (Kanai et al., 2010; Maniscalco and Lau, 2011).

We ran further analyses, assessing meta-d' separately for trials where the participants made a “yes” or a “no” response for the YN task. Based on the data from Experiment 3, we confirmed that meta d' for “yes” responses significantly correlated between visual and memory tasks (*r* = 0.2364, *p* = 0.0179), but this was not the case for meta d' for “no” responses (*r* = 0.1453, *p* = 0.1493). Therefore, it appears that the lack of correlations observed in our Experiment 3, as well as in our Experiment 2 and Baird et al. (2013), were due specifically to some idiosyncratic nature of metacognition after “no” responses in a YN task.

In general, it is known that metacognition after “no” responses may be less efficient (Kanai et al., 2010; Maniscalco and Lau, 2011). We found evidence supporting this in the memory task for both Experiment 2 and Experiment 3. In the memory YN task for Experiment 2, for which meta d' for “yes” responses was significantly greater than for “no” responses [the means were 1.511 and 0.990, respectively, *t*_(99)_ = −4.475, *p* < 0.001], and for Experiment 3 memory task (the means were 1.295 and 1.106 for meta-d' after “yes” and “no” responses, respectively), although the latter did not reach statistical significance [*t*_(99)_ = −1.255, *p* = 0.213]. Conversely for our Experiment 3 visual task, meta d' for “yes” responses was *lower* than for “no” responses (the means were 1.169 and 1,285, respectively), although the difference did not reach statistical significance [*t*_(99)_ = 0.594, *p* = 0.554].

Based on these results, we further tested the possibility that meta-d' for “no” responses is problematic for assessing cross-domain metacognitive correlations, only when it is lower than meta-d' for “yes” responses. In support of this possibility, we found that in Experiment 3, meta d' for “yes” responses on the memory task was significantly correlated with (overall) meta d' for the visual task in our Experiment 3 (*r* = 0.2131, *p* = 0.0333), meaning that including the metacognitive measure after “no” responses was not a problem for revealing metacognitive correlations. Likewise, in Experiment 2 we also tested for an association between meta d' for “yes” responses on the memory task and (overall) meta d' for the visual task and found a strong trend toward statistical significance (*r* = 0.1890, *p* = 0.0596). Further supporting the above suggestion, we found that meta d' for “yes” responses on the visual task was not significantly correlated with the overall meta d' for the memory task in Experiment 3 (*r* = 0.1640, *p* = 0.1031), where meta-d' for “no” responses was higher than meta-d' for “yes” responses.

In sum, it seems that including meta-d' for “no” responses is only limiting our ability to reveal across-domain metacognition when it is lower than meta-d' for “yes” responses. Below we further interpret this finding in terms of metacognitive mechanisms.

## Discussion

We showed that requiring 2AFC judgments for both a visual task and memory task allowed us to show a positive correlation between visual and memory metacognition (Experiment 1), whereas this correlation significantly decreased and ultimately disappeared when requiring 2AFC for one task and YN judgments for the other (Experiment 2). We also found no correlation when requiring YN judgments for both tasks (Experiment 3), suggesting that using the same task for both domains may not be necessary for observing cross-domain correlation in metacognitive sensitivity. Together, these results suggest that our failure to find a correlation in Experiments 2 and 3 resulted specifically from YN judgments introducing more ambiguity into the decision process than 2AFC. Further analyses indicated that, specifically, it is metacognition after “no” responses that may be the problem, especially in the memory task.

What is it specifically about “no” responses in the memory task that might have caused the observed results? One subtle difference between our YN memory and visual tasks is that the former is what has been called a “true” detection task, the goal of which is to detect a stimulus presence from a stimulus absence, whereas the latter is a “pseudo” detection task, in which one detects the presence of a stimulus *feature* while the absence case contains a similar level of stimulus energy despite the lack of that particular feature. This distinction was introduced by Maniscalco and Lau [Maniscalco and Lau (2011)], who reported that meta d' for “no” responses was lower than for “yes” responses on task in which the target-present condition involved the presence of some physical feature (solid dots), which carried additional stimulus energy.

However, meta d' for “yes” and “no” responses were similar on a “pseudo” detection task, in which the target-absent condition contained not just the absence of a certain physical feature, but the feature that was replaced by another feature (unfilled circles). Above we suggested that “no” judgments are somewhat more convoluted or difficult than “yes” judgments, because, in the absence of evidence, it may be difficult to assess certainty. If this is true, this may only apply to our true-detection, memory YN task, but not to the visual YN task, which is akin to “pseudo” detection. This is because, unlike in true-detection tasks, in our YN visual task subjects did not discriminate between the presence or absence of a stimulus; instead they judged whether a stimulus was, on average, big or small. Overall, the pattern of results supports the hypothesis that it is metacognition after a “no” responses in “true” detection that is causing the problem in limiting our ability to reveal metacognition across task domains.

Our results agree with other similar studies and may inform their interpretations. As in our Experiment 2 and Baird et al. (2013), both Baird et al. (2015) and Sadeghi et al. (2017) used YN judgments for their memory task (which were true detection tasks) and 2AFC judgments for their visual task and found no correlation between metacognitive sensitivity for visual perception and memory. As in our Experiment 3, Fitzgerald et al. (2017) used YN judgments for both their memory task (a true detection task) and visual task (a pseudo detection task) and, again, found no cross-domain correlation. The authors interpret these results as reflecting a genuine lack of correlation or a genuine difference between the task domains. However, the pattern of results of the present study may provide an alternative interpretation: given that metacognitive sensitivity was observed only when 2AFC judgments were used for both domains, but not observed as long as the memory task was a YN task, the absence of cross-domain correlation in metacognitive sensitivity in those studies could be attributed to the use of YN judgments in the memory task.

Several comparable studies also agree with our Experiment 1 and McCurdy et al. (2013). Samaha and Postle (2017) used multi-choice orientation estimation tasks, which are not 2AFC tasks but are certainly not genuine YN detection tasks either, and they found a positive correlation between visual and memory metacognitive performance. Faivre et al. (2017) used 2AFCs and found positive correlations for metacognitive efficiency across the visual, auditory, and tactile perceptual domains.

Despite the above agreement, one relevant finding conflicts with our results. As in our Experiment 1 and McCurdy et al. (2013) and Morales et al. (2018) required 2AFC judgments for both their visual and memory task. However, they found no significant correlation across domains for metacognitive efficiency. A likely explanation is that Morales et al. (2018), which included 24 subjects, was underpowered to detect an effect in comparison with our Experiment 1, which included 100 subjects. In support of this possibility, power analyses revealed that given the effect size found in our Experiment 1 with 100 subjects (before subject exclusion; effect size = 0.2470), with alpha set at < 0.05, power was only 0.2228 for *n* = 24. This means that even if the effect was actually there, it was unlikely to have been detected given the sample size, so a null finding is unsurprising.

Also, in somewhat of disagreement with our results here, Valk et al. (2016) used 2AFC for visual perception and multiple-choice questions with three response options for higher-order cognition and found no correlation across these domains. In another study, Garfinkel et al. (2016) used a variety of YN and two-choice tasks (e.g., heart-rate synchronicity detection, tactile grating orientation discrimination, inspiratory resistance detection; none of these were 2AFC tasks) and yet found a positive correlation (unlike in our Experiment 3) between cardiac and respiratory metacognition, but not between either of these domains and tactile metacognition. It is arguable that none of the tasks in Garfinkel et al. (2016) are “true” detection tasks but, given the results of Valk et al. (2016), it may also be the case that our proposed view regarding YN and 2AFC tasks here only applies to studies of metacognition for certain domains in perception and memory. Alternatively, it could be related to the above-mentioned issue of sample size and power.

One may be concerned about the visual YN task used in the present study, as participants needed to remember the examples of “medium” sizes in order to make YN judgments about the average sizes of the subsequently presented clusters of circles. This could make the visual YN task similar to a memory task. However, it should be noted that our visual and memory tasks were fundamentally different in terms of the dimensions on which decisions were made. In the visual YN task, decisions were based on size, which is a visual feature by definition. In both the 2AFC and YN memory tasks, decisions were based on whether a stimulus had been presented before, which is a memory representation by definition. Furthermore, many visual tasks (or, in general, perceptual tasks) involve some kind of memory, e.g., after a Gabor patch or a random-dot motion pattern has disappeared, the participant makes judgment about its orientation or motion direction. Involving memory in a visual task does not make a visual task “not visual,” as long as performance in the task still heavily relies on visual perception. For example, in the present study, even if a participant had perfect memory about the “medium” size, visual processes would still be involved in perceiving the size of the tested clusters of circles. Therefore, we believe our visual and memory tasks tapped on different processing domains.

One possible limitation about the present study is that our first two experiments were not direct replications of McCurdy et al. (2013) and Baird et al. (2013). One notable difference was that circle stimuli were used for both tasks in the present study, whereas gratings and words were used for the visual and memory tasks, respectively, in the previous studies. While in one sense this might be perceived as a limitation, matching the stimulus type across the tasks was meant to correct a potential confound in the previous studies (as already described), and it is therefore our opinion that it should be viewed as a strength rather than a limitation.

Another limitation may be that the experiments were conducted online, in which participants' identities or visual and mental conditions could not be verified in similar ways as in experiments conducted in laboratory settings. While this may be a concern for online research in behavioral science in general, it has been shown that (1) online participants produce largely comparable results in both cognitive and perceptual experiments as laboratory participants do (Germine et al., 2012; Crump et al., 2013), and (2) the impact of repeated participation of the same individual is minimal (Berinsky et al., 2012). Specifically, because devices and apparatus tend to be more stable in laboratory settings than in online settings, the contribution from “common-method” variances and/or biases to the variability of the data could be different between laboratory and online settings. However, given that both means and variances of performance were largely similar between online and laboratory settings (Germine et al., 2012), the common-method variances and/or biases may affect both online and laboratory experiments in similar way and to a similar extent. While we believe the benefit still outweighs the damage, especially for the purposes of the present study, we acknowledge that these concerns are valid for all online studies in general. It would be nice for a study similar to the present one to be conducted in laboratory settings in the future, so that one could analyze the effects of settings-related factors on metacognition.

To conclude, 2AFC and YN judgments were traditionally well-defined task procedures that were clearly distinguished from one another (Macmillan and Creelman, 2004). However, in recent years it has become increasingly common for researchers to label any two-choice discrimination tasks as 2AFC (Peters et al., 2016). This may be beyond a simple terminological issue, as the results of our experiments show that conflating 2AFC and YN judgements can lead to substantive consequences. Future studies should be more careful in this subtle, yet important issue.

Because meta-d' measures are also best suited for 2AFC tasks (Maniscalco and Lau, 2014), we suggest using genuine 2AFC tasks whenever possible. In most instances, this is easy to implement: for any two-choice task, we can modify it to present both stimuli in the same trial in temporal succession, or spatially one next to another, and ask the subject to identify the temporal or spatial arrangement of the pair, instead of to tell the identity of a single stimulus. Because one of the two stimuli can be made “blank,” a two-choice spatial localization task also counts as 2AFC (Macmillan and Creelman, 2004). Therefore, fortunately, the issues discussed here are easy to empirically address in future experiments.

## Ethics Statement

This study was carried out in accordance with the recommendations from the University of California Los Angeles (UCLA) Office of the Human Research Protection Program with written informed consent from all subjects. All subjects gave written informed consent in accordance with the Declaration of Helsinki. The protocol was approved by the UCLA Institutional Review Board.

## Author Contributions

All authors contributed conception and design of the study. ER and NG conducted the study, performed the statistical analyses, wrote the first draft of the manuscript. AL and HL contributed to critical revision of the manuscript, read and approved the submitted version.

### Conflict of Interest Statement

The authors declare that the research was conducted in the absence of any commercial or financial relationships that could be construed as a potential conflict of interest.
